# Trusting Generative AI for Health Advice: Preregistered Survey Experiment

**DOI:** 10.2196/97882

**Published:** 2026-06-22

**Authors:** Asheley R Landrum, Nitin Verma, Amanda Kehrberg

**Affiliations:** 1 Walter Cronkite School of Journalism and Mass Communication, Arizona State University, 555 N Central Ave, Phoenix, AZ, 85004-1248, United States, 1 602-496-5555; 2 School of Information Sciences, University of Illinois Urbana-Champaign, Champaign, IL, United States

**Keywords:** artificial intelligence, ChatGPT, digital health, health communication, health information seeking, medical skepticism, risk perception, trust

## Abstract

**Background:**

Generative artificial intelligence (AI) systems are increasingly used for health information seeking, yet it remains unclear how the public evaluates AI-generated health advice relative to guidance from credentialed clinicians in digital environments. Understanding the conditions under which AI is perceived as credible is critical as these systems become integrated into digital health ecosystems.

**Objective:**

This study examined how source type (a human nurse in an online portal, a health care–specialized generative artificial intelligence system framed as an “AI nurse,” or ChatGPT [OpenAI], a general-purpose chatbot), message characteristics, contextual risk, values framing, and individual differences in medical skepticism and experience with AI shape credibility evaluations of the provided advice and its purported source.

**Methods:**

In a preregistered online experiment, a national sample of US adult participants (N=1502) was randomly assigned to 1 of 3 source conditions and evaluated health advice across 3 scenarios: low risk (dietary advice for cholesterol), high risk (chest pain triage), and a morally sensitive scenario (egg freezing). Advice type (intuitive vs counterintuitive) was manipulated in the risk scenarios, and ideological framing (neutral, conservative-leaning, and liberal-leaning) was manipulated in the morally sensitive scenario. Primary outcomes included participants’ perceived credibility of the advice and beliefs about whether the patient should follow it. Source-level perceptions of competence and benevolence were also assessed. Medical skepticism and prior AI experience were examined as moderators.

**Results:**

Advice attributed to a human nurse was rated as more credible than advice attributed to either AI source. Message intuitiveness showed effects comparable to, and sometimes larger than, the effects of source: intuitive advice was perceived as more credible than counterintuitive advice, with this difference amplified in high-risk contexts. In the morally sensitive scenario, ideological framing influenced perceived bias but did not interact significantly with source. Medical skepticism moderated source evaluations: higher skepticism was associated with greater perceived competence of the AI nurse and lower perceived competence of the human nurse.

**Conclusions:**

Generative AI is evaluated within existing credibility frameworks rather than dismissed outright as inferior to human expertise. While licensed clinicians retain a credibility advantage, AI-generated advice is generally perceived as competent and legitimate. Importantly, individuals skeptical of traditional medical authority may evaluate AI-based guidance more favorably, suggesting that AI systems may redistribute—rather than uniformly erode—trust in health advice. As AI tools become embedded in patient-facing health platforms, message design and audience characteristics may shape acceptance more strongly than source labeling alone.

## Introduction

### Background

Generative artificial intelligence (AI) tools are increasingly embedded in everyday information-seeking practices, including domains traditionally reserved for credentialed experts [1]. Health advice is no exception, and a sizable proportion of people report turning to general-purpose AI tools, such as ChatGPT (OpenAI), for health-related inquiries [2]. Notably, platform developers are formalizing this use: OpenAI, for example, has announced a new tool, ChatGPT Health, stating that health-related information-seeking is one of ChatGPT’s most popular uses, with “hundreds of millions of people asking health and wellness questions each week” [3]. As these systems increasingly function as media for transmitting scientific knowledge to lay publics [4], they occupy a unique position within the health communication ecosystem: neither fully institutional nor fully interpersonal, neither a licensed professional nor an anonymous webpage [5]. Prior research has long established that trust in the source of health information is central to whether individuals act on medical advice [6-8]. Yet it is unclear how generative AI is evaluated when it offers health guidance: do people treat it as a credible source of information, and, if so, under what conditions?

### Epistemic Trust and Source Credibility

Epistemic trust is the willingness to accept information from another as reliable, relevant, and generalizable; that is, to treat the information source as a legitimate knower whose claims can guide belief and action [9-11]. Such trust is typically grounded in evaluations of source credibility—often conceptualized in terms of competence or expertise and benevolence or trustworthiness [12,13]. Indeed, decades of social science research demonstrate that people’s perceptions of source credibility influence whether and to what extent messages are accepted [14]. In science communication, trusting sources based on perceived expertise has been shown to influence public responses to scientific recommendations, particularly in domains involving uncertainty or risk [15]. In health contexts, trust in medical professionals predicts adherence to treatment recommendations, preventive behaviors, and willingness to seek care [16,17]. As new “actors” enter the health communication environment, understanding the circumstances under which patients deem messages and sources credible is essential for anticipating downstream effects on public health behavior.

Licensed medical professionals have traditionally occupied positions of epistemic authority [18]; research finds that physicians and nurses are typically perceived as very trustworthy, even amid declining trust in other types of experts and social institutions [19] (see also [20,21]). The clinical encounter itself is structured around asymmetries in knowledge, with patients typically deferring to experts to interpret symptoms, assess risks, and obtain recommendations for appropriate courses of action. Thus, we may assume that credentialed human providers presently serve as a benchmark of credibility against which other (nonhuman) sources ought to be evaluated.

At the same time, epistemic authority does not imply infallibility. Health care professionals, like other humans, are susceptible to cognitive biases, knowledge gaps, time constraints, and diagnostic errors. Patients’ lived experiences, including misdiagnosis, perceived dismissal, or inequitable treatment, may further complicate their assumptions of health care practitioners’ credibility. A trust deficit in this sense could reflect antiexpert sentiment, but it also may arise from an awareness of human fallibility and structural limitations within health care systems. Against this backdrop, generative AI systems that can digest and synthesize extensive medical literature may establish new benchmarks for evaluating the quality of advice. AI systems trained on expansive, peer-reviewed corpora may be perceived as less prone to individual bias, less influenced by interpersonal dynamics, and more consistently evidence-based than any single human provider is capable of being [22,23]. To the extent that patients equate credibility with comprehensiveness, neutrality, or data-driven reasoning, such systems could, at least in some contexts, be viewed as rivaling or even exceeding the perceived credibility of credentialed—but inherently human—experts.

Importantly, however, generative AI systems themselves are not monolithic. If patients evaluate human credibility in part by inferring competence and knowledge boundaries, then perceptions of AI expertise could also depend on cues that signal domain specialization [24]. Research on expertise heuristics demonstrates that audiences rely on markers such as credentials, role labels, institutional affiliation, and context to assess whether a source is qualified to speak on a given issue [25,26]. Use of such heuristics may also extend to AI agents [27,28]. A system described as a general-purpose chatbot may be evaluated differently than one framed as a custom-trained medical assistant or “AI nurse,” even when both rely on similar underlying architectures—and especially if users assume differences in training data, evidentiary standards, or scope of knowledge. Consistent with this logic, studies of algorithmic systems suggest that perceived domain fit and task alignment meaningfully shape trust in and compliance with AI recommendations [29]. Distinguishing between general-purpose AI tools and health care–specific systems enables us to examine whether and how specialization cues influence perceptions of credibility.

### Message Characteristics and Other Factors

Although source characteristics play an important role in credibility assessments, message features themselves can shape whether advice is accepted (and can even influence perceptions of the source of the information [13,30]). In particular, individuals tend to evaluate information more favorably when it aligns with their prior beliefs and expectations [31]. Dual-process theories of persuasion suggest that when individuals rely on heuristic processing, cues such as coherence, familiarity, and intuitive plausibility are influential [32,33]. For example, people may view accurate advice that contradicts their common sense–based expectations as less credible. In health contexts, where individuals hold lay theories (as opposed to expert knowledge) about diet, illness, and risk, recommendations that seem counterintuitive may face heightened skepticism, regardless of the source.

The perceived stakes of a decision may also shape credibility judgments. Research on risk perception demonstrates that individuals process information differently in low- versus high-risk contexts, with heightened sensitivity to potential threats amplifying reliance on heuristics and affective cues [34-37]. Research on the affect heuristic suggests that when consequences are perceived as severe or uncertain, individuals rely more heavily on gut reactions and intuitive evaluations than on analytic scrutiny [34,38]. In health domains, high-risk scenarios can trigger defensive processing [39,40], which may magnify skepticism toward advice that appears counterintuitive. If so, advice that violates lay expectations may incur a larger credibility penalty in higher-risk contexts than in lower-risk or routine ones.

Beyond risk and advice intuitiveness, credibility judgments may also be shaped by the extent to which proffered health advice activates personal values and identity. Research on motivated reasoning suggests that individuals evaluate information in ways that protect preexisting beliefs and moral commitments [41-45] (see also [31]). Health decisions involving reproduction and bodily autonomy, for example, may be evaluated in light of their own value orientations (particularly religiosity and political identity), rather than solely on medical grounds. When proffered advice signals alignment with particular values, it is likely to be perceived as biased, and such perceptions of bias may influence compliance and judgment of credibility independently of a source’s signaled expertise. However, as noted earlier, people may expect less bias from AI sources than from human sources [22,23,46,47]. Examining health advice in contexts intertwined with individuals’ ideological values and moral commitments, then, allows us to assess another context in which generative AI systems may be evaluated differently from human health care providers.

Individuals’ judgments about the credibility of health information providers also reflect their broader orientations toward medical authority. Medical skepticism, for instance, combines institutional distrust with confidence in one’s own ability to understand and manage health concerns [48]. Rather than rejecting medicine outright, participants with this orientation question the necessity of professional intervention, prefer self-directed decision-making, and treat personal experience as a meaningful source of health knowledge; and research links medical skepticism to unhealthy behaviors, lack of insurance, absence of a primary care provider, delayed or avoided physician visits, and limited use of preventive care [48]. Related work on institutional trust shows that individuals who distrust established authorities often discount expert recommendations, particularly when they perceive power or knowledge asymmetries [49]. At the same time, scholarship on self-efficacy and lay health expertise finds that people rely on personal judgment, informal networks, and their own experiences when evaluating medical information [50]. Together, these dynamics suggest that emerging technological advice sources—such as generative AI—may be evaluated more favorably by individuals who feel skeptical of traditional medical authority yet confident in their own interpretive capacities.

### This Study

Here, we investigate how generative AI is evaluated as a source of health advice, comparing perceptions when participants are told that advice comes from a licensed human nurse, an AI nurse, or a general-purpose chatbot (ChatGPT). Drawing on source credibility and epistemic trust theory, risk perception research, and motivated reasoning, we preregistered directional hypotheses regarding the source manipulation (hypotheses 1a-1f and hypothesis 4). We did not preregister hypotheses regarding potential main effects of the message characteristics (hypotheses 2 and 3).

First, we hypothesized that human sources would be perceived as more competent (hypothesis 1a) and benevolent (hypothesis 1b) than AI sources. Similarly, we predicted that advice attributed to a licensed human nurse would be perceived as more credible (hypothesis 1c) and more worthy of adherence (hypothesis 1d) than advice attributed to either AI source (consistent with relevant work by Reis et al [51]). Second, based on motivated reasoning and dual-processing theories, we expected that (hypothesis 2a) intuitive advice would be judged as more credible than counterintuitive advice, with this difference amplified in high-risk contexts (an intuitiveness-by-risk-level interaction, hypothesis 2b). Third, in the morally sensitive scenario, we anticipated that (hypothesis 3) ideological framing would shape perceptions of advice bias and credibility, and we specifically expected (hypothesis 4) the AI sources to be perceived as less biased than the human nurse. Finally, informed by scholarship on epistemic trust, motivated reasoning, and perceptions of algorithmic objectivity, we explored (research question 1 [RQ1]) whether individual differences in (1) medical skepticism and (2) experience with AI tools moderate credibility evaluations of human and AI sources.

## Methods

### Ethical Considerations

The study was approved and categorized as exempt by the Arizona State University Institutional Review Board on October 3, 2024 (STUDY00020970). In accordance with best practices for the ethical and transparent reporting of internet-based surveys, this study adhered to the CHERRIES (Checklist for Reporting Results of Internet E-Surveys) guidelines [52]. Refer to Checklist 1. No personal or identifying information was collected or stored. Our methods, hypotheses, and analysis plan were preregistered on the Open Science Framework on June 14, 2025, 1 month prior to data collection [53].

### Participants

Participants (N=1502) were recruited via the Cint panel to approximate US national demographics. Our sample was 74% (1111/1502) White, 14% (208/1502) Black or African American, 6% (78/1502) Asian American or Pacific Islander, 2% (32/1502) American Indian or Alaska Native, and 6% (72/1502) other or nonresponse; 13% (190/1502) identified as Hispanic or Latino. Participants were 54% (807/1502) female and ranged in age from 18 to 99 (mean 47, SD 18) years, with regional distribution spanning the Northeast (18%, 271/1502), Midwest (22%, 329/1502), South (40%, 595/1502), and West (20%, 306/1502). The sample size was determined using a power analysis in R (R Foundation for Statistical Computing) for our preregistered hypotheses (assuming a medium effect size and 80% power) as well as the available amount of funds to pay for participant recruitment.

### Design and Procedure

We conducted a survey experiment, built using Qualtrics (Qualtrics LLC) survey software, with data collection between July 14 and 15, 2025. We requested 1500 participants sampled to match US Census data (for adults aged 18 years and older) from the Cint panel, which recruited potential participants and provided them with a link to our survey. After opening the survey link, reading the study description, and voluntarily consenting to participate, participants were randomly assigned (via a randomizing function in the survey software) to 1 of 3 source conditions: a licensed human nurse, a health care–specialized “AI nurse,” or ChatGPT. To preserve data quality, individuals who started the study but failed an attention check requiring them to correctly identify the source of the advice in the study (ie, their source condition) were immediately dropped (they were not allowed to complete the rest of the survey) and their data were deleted. Thus, these individuals are not a part of the final sample of 1502 participants. Participants were compensated by Cint in line with their survey panel agreements. Median completion time was 11.97 (IQR 11.32) minutes.

Each participant read and answered questions about 3 scenarios that were presented in a randomized order: a low-risk scenario (whether to eat eggs when trying to reduce cholesterol), a high-risk scenario (whether to go to the emergency room for chest discomfort), and a morally sensitive (or values-relevant) scenario (whether to freeze one’s eggs to preserve fertility for later in life). Each scenario showcased a conversation between a fictional person seeking health advice and the source of the advice. Scenario topics were chosen based on our unpublished pilot study. The full texts are available in Multimedia Appendix 1 and on our Open Science project page [54].

Our design included 2 substudies. Refer to Table 1. Substudy 1 (the low- and high-risk scenarios) used a mixed design with source (human nurse, AI nurse, or ChatGPT) and advice intuitiveness (intuitive or counterintuitive) as between-subjects factors and scenario risk level (low-risk and high-risk) as a within-subjects factor. Substudy 2 (the morally sensitive scenario) used a fully between-subjects design crossing source with advice bias (neutral, conservative-leaning, or liberal-leaning).

**Table 1.** Study design and sample size^a^.

Substudy		Source (between-subjects)		
	Scenarios (within-subjects)	ChatGPT (n=487)	AI^b^ nurse (n=527)	Human nurse (n=488)
Substudy 1	Low-risk scenario (dietary advice) High-risk scenario (chest pain)	Advice intuitiveness: Intuitive (n=253) OR Counterintuitive (n=234)	Advice intuitiveness: Intuitive (n=261) OR Counterintuitive (n=266)	Advice intuitiveness: Intuitive (n=241) OR Counterintuitive (n=247)
Substudy 2	Morally sensitive scenario (egg freezing)	Advice bias: Neutral (n=147), Liberal (n=168), OR Conservative (n=172)	Advice bias: Neutral (n=165), Liberal (n=179), OR Conservative (n=183)	Advice bias: Neutral (n=173), Liberal (n=153), OR Conservative (n=162)

a Substudy 1 (source × advice intuitiveness × risk level) applied only to the low- and high-risk scenarios, whereas Substudy 2 (source× advice bias) applied only to the morally sensitive scenario. Scenario order was randomized between participants. The total sample size was 1502.

b AI: artificial intelligence.

After each scenario, participants rated advice credibility, the perceived ideological bias of the advice (perceived bias), and their agreement that the character in the scenario should follow the advice (take advice). After completing the scenarios, participants evaluated their perceptions of the source’s competence and benevolence and completed measures of medical skepticism, religiosity, prior generative AI experience, and other demographics.

### Manipulations and Measures

As stated above, we used 3 between-subjects manipulations. First, the source of health advice (ie, source) was randomly assigned and held constant across all three scenarios presented to a participant: (1) a licensed registered nurse (called “Nurse Dobson”) responding via an online patient portal; (2) an AI nurse—a custom-trained generative AI designed to simulate a health care professional using medical data; or (3) ChatGPT—a general-purpose AI developed by OpenAI that can provide information across domains, including health, but is not purpose-built for clinical use. Second, advice intuitiveness (substudy 1) was randomly assigned (using the survey software’s randomizing tools) as either intuitive (advice consistent with common expectations; eg, reducing saturated fat to lower cholesterol; seeking immediate emergency care for chest discomfort) or counterintuitive (advice that departs from common expectations but is accurate; eg, endorsing full-fat dairy while reducing carbohydrates; recommending acid-reflux management when a heart attack is unlikely based on contextual factors). Advice intuitiveness also remained consistent for each participant across both the low-risk and high-risk scenarios (the within-subjects manipulation in substudy 1); it was not relevant to the morally sensitive scenario. Third, in the morally sensitive scenario only (substudy 2), we manipulated advice bias between-subjects as neutral (medically focused and balanced framing), conservative-leaning (prioritizing starting a family and natural means of conception), or liberal-leaning (emphasizing reproductive autonomy and individual empowerment). The exact wording of each message appears in the supplementary materials on our Open Science Framework project page [54].

Advice credibility was measured with a single-item Likert-type scale asking, “How would you rate the credibility of the advice [PATIENT] received from [SOURCE]?” (1=not at all credible to 5=extremely credible). Belief that the patient should follow the advice was also assessed with a single item, “In your view, should [PATIENT] take the advice given by [SOURCE]?” (1=definitely no to 4=definitely yes). Both items were asked after each of the 3 scenarios. Perceived ideological bias of the advice was measured using a single item with a directional scale (“Do you think the advice [PATIENT] received from [SOURCE] is politically biased?”) ranging from 1=strong right-wing or conservative bias to 5=strong left-wing or progressive bias, with 3=no, I don’t think it is politically biased and 99=other (please specify) followed by a textbox for open-ended responses. The 35 participants who selected “Other” were list-wise deleted from analyses using this variable, and the vast majority of these participants wrote “none,” “not sure,” or “I have no idea” in the textbox.

Two multiitem scales—one to measure perceived source competence and the other to measure perceived source benevolence—assessed source credibility after all 3 scenarios were presented. Perceived source competence was measured using five 7-point semantic differential items (experienced-inexperienced, expert-inexpert, competent-incompetent, poorly trained–well trained, and informed-uninformed [12,55]). These items showed good interitem reliability, with Cronbach α=0.84 (95% CI 0.83-0.86). Perceived benevolence was measured with four 7-point semantic differential items (eg, trustworthy-untrustworthy, likely to tell the whole story–unlikely to tell the whole story, biased-unbiased, and likely to separate fact and fiction–unlikely to separate fact and fiction [12,55]). The perceived benevolence items had weaker interitem reliability than the perceived source competence items (Cronbach α=0.66, 95% CI 0.63-0.69). As specified in our preregistration, we used the factor scores extracted from a graded response model (item response theory analysis [56]) in our analyses.

Several individual difference measures were also included for exploratory analyses. Medical skepticism was measured with 4 items from prior research [48]: “I understand my health better than most doctors do,” “I can overcome most moderate or severe illnesses without help from a medically trained professional,” “home remedies are often better than those prescribed by a doctor,” and “if I get sick, it is my own behavior that determines how soon I get well again” (Cronbach α=0.71, 95% CI 0.68-0.73). These items reflect both doubt about the usefulness of medical intervention and higher self-efficacy in handling illness. We also measured participants’ religiosity by averaging across 2 items (each centered and scaled). The first question asked participants how much guidance their faith, religion, or spirituality provides them in their day-to-day life (on a 6-point ordinal scale). The second question asked participants how frequently they pray (on a 5-point ordinal scale). We also asked participants how much experience they have using generative AI tools such as ChatGPT, DALL-E (OpenAI), Gemini (Google LLC), and so on. We provided 6 response options: no experience (29%, 433/1502), a little experience (“I’ve tried them once or twice,” 23%, 350/1502), some experience (“I use them occasionally,” 25%, 374/1502), a lot of experience (“I use them regularly for work, school, or personal tasks,” 15%, 227/1502), expert-level experience (“I use or develop generative AI tools frequently and understand how they work,” 4%, 62/1502), and other (please specify, n=6). Participants who selected “other” were list-wise deleted from analyses using this variable. This item was included because recent studies have shown that one of the strongest predictors of trust in generative AI technology is experience using that type of technology [57]. These items were in addition to standard demographic variables collected (eg, age, education, income, and gender).

## Results

### Overview

Our primary aim was to examine whether the perceived source of health advice, human nurse, AI nurse, or ChatGPT, influences participants’ perception of the credibility of that advice and whether the advice should be taken by the person described in a given scenario (hypothesis 1). Second, we were interested in whether that trust (and/or differences in trust) varied based on health context (eg, scenario characteristics: hypothesis 2: intuitiveness of the advice given and level of risk inherent in the scenario; hypothesis 3: advice bias) and audience characteristics (eg, political views, prior experience with AI, and medical skepticism; RQ1a and RQ1b). A summary table indicating each hypothesis and whether it was supported by the findings is provided in Multimedia Appendix 2.

### Substudy 1: Risk Level and Intuitiveness of Advice

For substudy 1, we had 2 outcome variables of interest: perceptions of the advice’s credibility (advice credibility) and the belief that the character in the scenario should take the advice (take advice). We analyzed the data in R using mixed-design ANOVAs (one model per outcome variable) and report the results in Table 2 below.

**Table 2.** Results from substudy 1^a^.

Outcome variable	Advice credibility			Take advice		
	*F* test (*df*)	*P* value	η_ρ_²	*F* test (*df*)	*P* value	η_ρ_²
Source	21.37 (2, 1494)	<.001	0.030	9.28 (2, 1491)	<.001	0.010
Advice intuitiveness (intuitiveness)	40.44 (1, 1494)	<.001	0.030	76.52 (1, 1491)	<.001	0.050
Source× intuitiveness	0.81 (2, 1494)	.45	0.001	0.06 (2, 1491)	.94	<0.001
Scenario risk level (risk)	7.41 (1, 1494)	.007	0.005	15.31 (1, 1491)	<.001	0.010
Source× risk	0.74 (2, 1494)	.48	<0.001	0.48 (2, 1491)	.62	<0.001
Intuitive× risk	18.54 (1, 1494)	<.001	0.010	32.17 (1, 1491)	<.001	0.020
Source× intuitiveness×risk	2.33 (2, 1494)	.10	0.003	0.04 (2, 1491)	.96	<0.001

a Source and advice intuitiveness (intuitive and counterintuitive) were the between-subjects variables, and scenario risk level (low risk and high risk) was the within-subjects variable.

When examining perceptions of advice credibility, there was a main effect of source, and follow-up tests with a Bonferroni correction found that advice from the human nurse (estimated marginal mean, ie, emmean 3.53, SE 0.05, 95% CI 3.44-3.63) was rated as more credible than that from the AI nurse (emmean 3.13, SE 0.05, 95% CI 3.04-3.22; *P*<.001) and ChatGPT (emmean 3.21, SE 0.05, 95% CI 3.11-3.30; *P*<.001), supporting hypothesis 1c. There was no significant difference in perceptions of advice credibility between the two AI sources (*P=*.68). We also found differences in advice intuitiveness and scenario risk level (Table 2). Counterintuitive advice (emmean 3.12, SE 0.04, 95% CI 3.04-3.19) was seen as less credible than intuitively correct advice (emmean 3.46, SE 0.04, 95% CI 3.39-3.54; *P*<.001), and advice given in the low-risk scenario (emmean 3.33, SE 0.03, 95% CI 3.27-3.39) was seen as more credible than advice given in the high-risk scenario (emmean 3.24, SE 0.03, 95% CI 3.18-3.31; *P=*.006).

**Figure 1.**
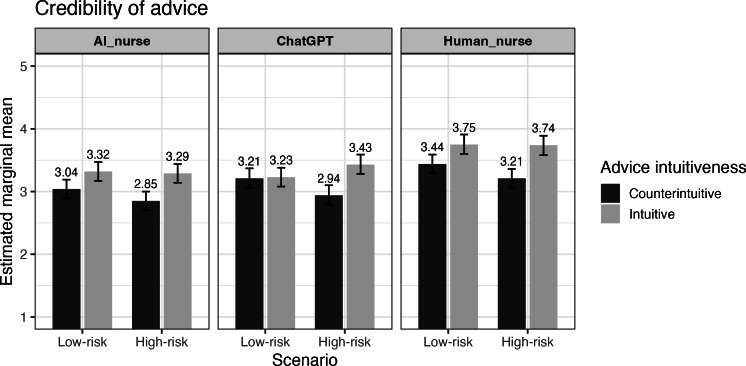
Estimated marginal means for advice credibility ratings broken down by source, advice type, and scenario (advice credibility was measured on a scale of 1 (not at all credible) to 5 (extremely credible), and a rating of 3 indicated moderate credibility).

The 3-way interaction between source, advice intuitiveness, and scenario risk level was only marginally significant (*P=*.10), whereas the 2-way interaction between advice intuitiveness and scenario risk level was statistically significant (*P*<.001), supporting hypothesis 2 (Table 2). Participants, averaged across the source conditions, perceived the counterintuitive advice as less credible than the intuitive advice, and the difference in perceived advice credibility between the counterintuitive and intuitive advice was greater for the high-risk scenario (difference estimate= –0.48) than for the low-risk one (difference estimate= –0.20). Refer to Figure 1.

The results for participants’ level of agreement that the scenario character should take the advice (ie, take advice) follow the same pattern as the results for participants’ perceptions of advice credibility. Refer to Table 2. Supporting hypothesis 1, participants who saw advice from the human nurse (emmean 3.36, SE 0.03, 95% CI 3.30-3.42) agreed more strongly that the scenario character should take that advice than participants who saw advice from an AI nurse (emmean 3.20, SE 0.03, 95% CI 3.15-3.26; *P*<.001) or ChatGPT (emmean 3.22, SE 0.03, 95% CI 3.16-3.28; *P*=.002). The difference between the AI nurse and ChatGPT was not significant (*P*=.89).

We also found a significant effect of advice intuitiveness (counterintuitive vs intuitive) on take advice that does not vary based on source (no significant advice intuitiveness by source interaction, *P*=.94). Overall, people agree more strongly that the scenario character should take the intuitive advice (emmean 3.40, SE 0.02, 95% CI 3.36-3.45) than the counterintuitive advice (emmean 3.12, SE 0.02, 95% CI 3.07-3.16; *P*<.001). There is also a significant effect of scenario risk level: participants agree more strongly that advice in the low-risk scenario is worth taking (emmean 3.31, SE 0.02, 95% CI 3.27-3.34) than advice in the high-risk scenario (emmean 3.22, SE 0.02, 95% CI 3.18-3.26; *P*<.001), and this, too, does not vary by source (no significant scenario risk level by source interaction, *P*=.62).

Supporting hypothesis 2, we found the expected significant interaction between advice intuitiveness and scenario risk level (Figure 1), such that participants, averaged across source conditions, perceived the counterintuitive advice as less credible than the intuitive advice, and the difference in take advice between the counterintuitive and intuitive advice types was greater for the high-risk scenario (difference estimate= –0.41) than for the low-risk one (difference estimate=–0.16; *P*<.001).

### Substudy 2: Morally Sensitive Scenario and Ideological Bias of Advice

For substudy 2 (hypotheses 3 and 4), in addition to evaluating perceived advice credibility and taking advice, we also evaluated whether, and to what extent, source, advice bias, and individuals’ own political ideology (ie, vote) influenced their perceptions of bias in the advice (ie, perceived bias). We tested these questions with a fully between-subjects ANOVA in R. Refer to Table 3. In the perceived bias analysis, we found no significant effects except for our advice bias manipulation (supporting hypothesis 3). Follow-up paired-samples *t* tests with Tukey correction showed that as intended, the conservative advice bias manipulation (mean 2.80, SD 0.96) was perceived as more conservatively biased than the liberal (mean 3.12, SD 0.90; Cohen *d*=0.34; *P*<.001) and neutral advice manipulations (mean 3.03, SD 0.87; Cohen *d*=0.25; *P*<.001), though the liberal advice did not differ significantly from the neutral advice (Cohen *d*=0.10; *P*=.27). Notably, the median response option across all 3 conditions was 3, which corresponded to “No, I don’t think it’s politically biased.”

**Table 3.** Results from substudy 2^a^.

	Advice credibility			Take advice			Perceived bias		
	*F* test (*df*)	*P* value	η_ρ_²	*F* test (*df*)	*P* value	η_ρ_²	*F* test (*df*)	*P* value	η_ρ_²
Source	12.47 (2, 1474)	<.001	.020	7.11 (2, 1472)	<.001	.009	0.95 (2, 1440)	.39	.001
Advice bias (bias)	11.93 (2, 1474)	<.001	.020	19.11 (2, 1472)	<.001	.030	15.98 (2, 1440)	<.001	.020
Vote	1.04 (2, 1474)	.36	.001	0.12 (2, 1472)	.89	<.001	0.06 (2, 1440)	.95	<.001
Source× bias	*0.78 *(4, 1474)	.54	.002	0.37 (4, 1472)	.83	.001	0.54 (4, 1440)	.71	.001
Source× vote	1.29 (4, 1474)	.27	.004	1.77 (4, 1472)	.13	.005	1.34 (4, 1440)	.26	.003
Bias × vote	0.09 (4, 1474)	.98	<.001	1.45 (4, 1472)	.22	.004	0.40 (4, 1440)	.81	.001
Source× bias×vote	0.24 (8, 1474)	.98	.001	0.79 (8, 1472)	.61	.004	1.41 (8, 1440)	.19	.008

a These ANOVA use type I sums of squares.

When evaluating advice credibility and taking advice, we found an effect of advice bias. Conservatively biased advice was seen as less credible (mean 3.06, SD 1.23) than neutral advice (mean 3.42, SD 1.17; Cohen *d*=0.30; *P*<.001) and liberally biased advice (mean 3.34, SD 1.22; Cohen *d*=0.23; *P*<.001). Participants agreed more strongly that neutral advice (mean 3.37, SD 0.74; Cohen *d*=0.43) and liberally biased advice (mean 3.28, SD 0.74; Cohen *d*=0.34) should be taken than conservatively biased advice (mean 3.08, SD 0.81; both *P* values <.001).

### Source Credibility

We modeled the relationships among source credibility (assessed by perceived source competence and benevolence), source, individual characteristics (basic demographics, voting behavior, and religiosity), prior experience with large language model–driven chatbots, and medical skepticism using a generalized linear model. As we wrote in our preregistration, this examination of the potential influence of individual differences variables on perceptions of competence and benevolence is exploratory. Refer to Table 4.

**Table 4.** Results of generalized linear model analyses predicting competence and benevolence perceptions^a^.

Outcome variable	Competence			Benevolence		
	β	*P* value	lmg (%)	β	*P* value	lmg (%)
Source (AI^b^ nurse vs ChatGPT)	0.04	.55	6.53	0.03	.52	5.05
Source (AI nurse vs human nurse)	0.56	<.001		0.49	<.001	
GenAI^c^ experience	0.11	.02	0.47	0.11	.02	0.23
Medical skepticism	0.16	<.001	0.06	0.02	.61	0.37
Age	0.17	<.001	2.04	0.18	<.001	2.55
Income	0.04	.14	0.29	0.02	.40	0.10
Gender	0.06	.23	0.09	0.15	.006	0.51
Vote (democrat vs other)	–0.04	.51	0.16	–0.04	.52	0.11
Vote (democrat vs republican)	–0.01	.92		–0.05	.46	
Religiosity	0.05	.048	0.36	0.04	.12	0.23
Education	–0.01	.74	0.12	–0.02	.56	0.09
Source (AI nurse vs ChatGPT) × medical skepticism	–0.21	<.001	1.04	–0.10	.10	0.48
Source (AI nurse vs human nurse) × medical skepticism	–0.22	<.001		–0.17	.01	
Source (AI nurse vs ChatGPT) × GenAI experience	0.06	.34	0.43	–0.03	.60	0.03
Source (AI nurse vs human nurse) × GenAI experience	–0.10	.11		–0.04	.54	

a Standardized regression coefficients and relative importance using the lmg metric (which partitions model *R*² into predictor contributions averaged across all possible orderings).

b AI: artificial intelligence.

c GenAI: generative artificial intelligence.

When predicting both competence and benevolence, we found a significant effect of source where the AI nurse (mean competence score [M_comp_] 4.82, SD 1.39; mean benevolence score [M_bene_] 4.76, SD 1.25) is perceived as significantly less competent and benevolent than the human nurse (M_comp_ 5.6, SD 1.30; Cohen *d*=0.58; *P*<.001; M_bene_ 5.39, SD 1.30; Cohen *d*=0.50; *P*<.001). Similarly, ChatGPT was seen as less competent (M_comp_ 4.88, SD 1.34; M_bene_ 4.81, SD 1.24) than the human nurse (both *P* values <.001, both Cohen *d* values >0.40). There was no significant difference between the two AI sources (both *P* values >.29).

It is worth noting, however, that all sources were generally considered credible, with average ratings for both competence and benevolence above the midpoint of the scale (4). Generative AI experience also contributed to greater perceptions of competence and benevolence regardless of source. Interestingly, being older was associated with having greater perceptions of both competence and benevolence, averaged across source (Table 4).

**Figure 2.**
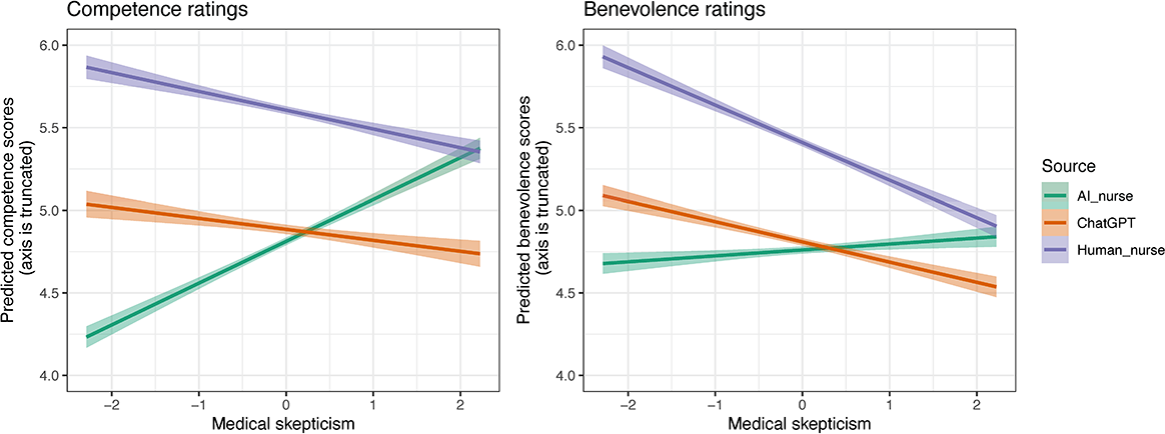
Relationships between medical skepticism and perceptions of source competence and benevolence by source condition (AI nurse, ChatGPT, and human nurse).

Most interestingly, we found a significant interaction between source and medical skepticism, such that the relationship between participants’ medical skepticism scores and their perceptions of competence and benevolence differs when rating the AI nurse versus the human nurse or ChatGPT. For example, as medical skepticism increases, perceptions of the AI nurse’s competence increase, whereas perceptions of the human nurse’s competence decrease. Refer to Figure 2. Simple effects correlation tests show that despite differences between the sources, the relationship between medical skepticism and credibility ratings is significant only for the AI nurse when rating competence and for the human nurse when rating benevolence. Refer to Table 5.

**Table 5.** Post hoc simple effects tests^a^.

Medical skepticism with	Competence		Benevolence	
	*r*	*P* value	*r*	*P* value
Human nurse	–0.08	.10	–0.13	.005
AI nurse	0.18	<.001	0.03	.44
ChatGPT	–0.04	.41	–0.08	.08

a Correlations between competence ratings or benevolence ratings and medical skepticism are shown for each source condition.

## Discussion

### Findings Summary

This study examined how generative AI tools are evaluated as sources of health advice relative to a licensed human nurse, and how those evaluations vary across message characteristics, contextual risk, and individual orientations toward medical authority (ie, medical skepticism). Overall, the findings largely supported the hypotheses. Consistent with hypothesis 1, human nurses were perceived as more competent and benevolent than either AI source, and advice was perceived as more credible and more worthy of being followed when it was attributed to the human nurse than either AI source. Supporting hypothesis 2, intuitive advice was judged as more credible than counterintuitive advice, and this difference was amplified in high-risk contexts. Supporting hypothesis 3, ideological framing influenced perceptions of bias and credibility in the morally sensitive scenario. Hypothesis 4 was not supported, as AI-generated advice was not perceived as less politically biased (in either direction) than advice from the human nurse. Finally, exploratory analyses related to RQ1 showed that medical skepticism moderated source evaluations, such that higher levels of medical skepticism were associated with greater perceived competence of the AI nurse, whereas the opposite pattern emerged for perceptions of the human nurse’s competence. In contrast, prior AI experience generally increased positive evaluations of all sources rather than differentially benefiting AI systems. Refer to Multimedia Appendix 2.

no our knowledge, this is among the first preregistered experiments to directly compare perceptions of advice credibility that purportedly comes from a human nurse, a domain-specialized AI system (“AI nurse”), or a general-purpose chatbot (ChatGPT), although we are aware of one paper that has examined perceptions of AI involvement in health advice (ie, [51], which compared an AI chatbot, a human physician, and a human physician collaborating with AI) and additional papers that have examined participants’ evaluations of advice that actually comes from physicians versus AI tools [58,59].

### Discussion of Key Findings

Several of our findings are particularly relevant for understanding how AI-mediated systems may reshape digital health ecosystems.

#### Message Characteristics Outweigh Source in Shaping Trust in Health Advice

Consistent with our first hypothesis [51], advice attributed to a licensed human nurse was rated as more credible and more worthy of taking than advice attributed to either AI source. However, these source effects were modest relative to message characteristics. Across all 3 sources, participants seemed to rely more on the intuitiveness of the advice itself. Intuitive recommendations were perceived as more credible than counterintuitive recommendations, particularly in high-risk contexts. These findings suggest that users are not treating AI-generated health advice as categorically distinct from human-delivered advice. Instead, they appear to apply similar advice-credibility heuristics—particularly whether the advice aligns with their expectations—to both human and artificial sources.

For digital health implementation, this pattern has important implications. As generative AI tools become integrated into patient portals, telehealth systems, and direct-to-consumer health platforms, developers and health systems may assume that labeling advice as AI-generated will meaningfully shift trust (up or down). Our results suggest that message content may exert a stronger influence than the source alone. In particular, counterintuitive but evidence-based recommendations may require additional explanation or scaffolding, especially in high-stakes scenarios where affective and heuristic processing are heightened.

#### Specialization Framing Failed to Boost Trust in AI Nurse Compared to ChatGPT

It is also noteworthy that we found no overall differences between ChatGPT and the AI nurse, despite anticipating that participants would be sensitive to the AI nurse’s description as a custom system trained on credible medical data and would use this as a cue of domain-specific expertise. There are several possibilities for this lack of difference. First, our design was between-subjects, and participants evaluated each source in isolation rather than engaging in relative judgment, which may have attenuated any influence of subtle framing differences. Second, ChatGPT, as a widely recognized and frequently used tool, may benefit from reputational credibility or the mere exposure effect, whereas the AI nurse—though described as specialized—was novel and thus lacked an established mental model. Third, participants may already perceive general-purpose AI systems as broadly competent across domains, including health, particularly given the growing visibility of AI-assisted information seeking. In this sense, describing ChatGPT as “not purpose-built” for health may not have been sufficient to meaningfully diminish perceived expertise, especially in the absence of explicit cues about potential limitations or risks (eg, the possibility of error or hallucination).

#### Medical Skepticism Associated With AI Nurse Competence but Not Human Nurses or ChatGPT

In our view, the interaction between medical skepticism and source credibility evaluations represents the most theoretically and practically consequential finding. Individuals higher in medical skepticism perceived the AI nurse as more competent, whereas similar individuals perceived the human nurse and ChatGPT as less competent. Medical skepticism reflects not only institutional distrust but also heightened confidence in one’s ability to manage one’s own health concerns [48]. For these individuals, generative AI may serve as a more acceptable alternative. Unlike licensed clinicians, AI systems are not (currently) embedded within visible institutional hierarchies, professional guilds, or regulatory structures. Furthermore, people often perceive computational systems as more objective, data-driven, and free from interpersonal bias than human experts [46,47], though some recent research has found participants to be suspicious of AI involvement in medical advice [51]. A perception of mechanical neutrality can foster trust, particularly in contexts where human institutions are viewed as politicized, self-interested, or fallible. At the same time, interacting with generative AI may afford users a heightened sense of agency and self-efficacy. Because individuals actively formulate prompts, evaluate responses, and iteratively refine queries, AI-generated guidance may feel less like externally imposed expertise and more like the product of one’s own information seeking. Research on online health information seeking and eHealth literacy suggests that independently sourcing and interpreting medical information can increase subjective knowledge, perceived control, and self-efficacy [60-62]. More broadly, self-determination theory and related work on autonomy suggest that when individuals perceive themselves as the originators of a decision process, they may be more likely to view its outcomes as legitimate [63]. In this way, AI systems may enable users to experience medical guidance as something they have “figured out” themselves, aligning more closely with values of autonomy and self-reliance. Consequently, individuals who question the legitimacy or authority of medical institutions may evaluate AI-based guidance not merely as a substitute for expertise, but as a different—and potentially more impartial and self-endorsed—source of knowledge.

That said, this was the one finding where ChatGPT and the AI nurse were evaluated differently. When it came to source competence, medical skepticism was positively associated with perceptions of the AI nurse’s competence (*r*=0.18, *P*<.001), but it was not significantly associated with perceptions of ChatGPT’s competence (*r*=–0.04, *P*=.41). The relationship between medical skepticism and perceptions of a source’s benevolence or trustworthiness did not significantly differ between the AI nurse and ChatGPT. One explanation for this divergence lies in how medical skepticism might reorient credibility judgments. As discussed, medical skepticism reflects not only distrust in institutionalized medical authority but also greater confidence in one’s own ability to evaluate health information. For these individuals, sources that preserve autonomy while still signaling relevant expertise may be especially appealing. The AI nurse uniquely satisfies both criteria: it carries cues of domain-specific medical knowledge while remaining outside traditional institutional hierarchies. In contrast, ChatGPT—framed as a general-purpose system—offers a similarly noninstitutional source but lacks clear signals of medical specialization. As a result, it may not meet the threshold for perceived competence in a health context, even among those predisposed to question traditional expertise.

This interpretation is consistent with research showing that audiences rely on domain-fit heuristics when evaluating expertise and that perceptions of credibility depend on whether a source is seen as appropriately qualified for a given task [24,28,29]. It also aligns with work suggesting that AI systems may be perceived as more impartial or less socially biased than human experts, which could elevate baseline perceptions of benevolence across AI sources [22,46,47]. Indeed, both AI sources may already be viewed as relatively neutral or non–self-interested, limiting differentiation on benevolence. In contrast, competence judgments appear more sensitive to the presence or absence of domain-specific expertise cues, possibly producing the observed divergence between the AI nurse and ChatGPT.

Importantly, this pattern suggests that medical skepticism does not simply increase or decrease trust in AI broadly. Rather, it appears to redistribute trust toward sources that combine perceived expertise with independence from traditional medical authority. In this way, generative AI systems that are framed as domain-specialized—but not institutionally embedded—may be particularly well positioned to gain credibility among individuals who are skeptical of conventional health care systems.

### Limitations

As with most research, this study has limitations. First, the AI nurse condition relied on descriptive framing to signal health care specialization. Participants may have inferred differences in training data, regulatory oversight, or institutional affiliation that were not explicitly specified. Future research could disentangle the effects of domain specialization cues from assumptions about accountability and evidentiary grounding. Second, our manipulation of ideological bias in the morally sensitive scenario was intentionally subtle, and median responses indicated that most participants did not perceive the advice as particularly politically biased (though they did perceive differences between conservative and liberal biased advice). Stronger or more explicit partisan cues may yield different credibility perceptions. Third, the study relied on hypothetical vignette scenarios, which necessarily simplify real-world health decision-making contexts. Although vignettes provide important methodological advantages, such as experimental control, standardized exposure across conditions, and the ability to examine sensitive or high-risk scenarios without requiring participants to disclose personal medical information, participants’ responses to hypothetical scenarios may differ from reactions in real clinical situations. In particular, evaluations of health advice may change when individuals are personally experiencing fear, uncertainty, pain, or urgency, or when they have an ongoing relationship with a health care provider or AI system. Relatedly, the selected scenarios focused on a limited number of contexts; evaluations may differ in chronic disease management, mental health, or pediatric settings where relational continuity and affective trust may be more central. Finally, although our counterintuitive advice conditions involved counterintuitive recommendations, we did not examine responses to clearly incorrect AI guidance, which may produce different patterns of trust and source differentiation.

### Conclusion

People are not abandoning human clinicians in favor of generative AI. Instead, AI appears to be yet another potential source of health advice that people evaluate using many of the same credibility judgments they apply to human sources, including perceptions of the source’s competence or expertise and benevolence or trustworthiness, characteristics of the advice itself (such as whether it seems intuitively plausible), contextual factors such as perceived risk, and through the lens of the individual’s orientations toward medical authority and institutional trust. Although licensed clinicians retain a credibility advantage, our study demonstrates that AI-based systems are nonetheless viewed as competent and legitimate sources of advice, particularly among individuals with prior AI experience and those skeptical of traditional medical authority. These patterns suggest that, rather than simply reducing trust in medical expertise overall, AI may change how people allocate trust across different sources of health information, with some individuals relying more heavily on AI systems alongside—or, in some cases, instead of—traditional health care providers. As AI tools become increasingly embedded in patient-facing health platforms, future research should examine how these evaluative processes operate in real clinical interactions and over time, particularly in a wider variety of contexts. Our findings also suggest that health communication interventions and AI system design should focus not only on informational accuracy but also on how message framing, transparency, and audience characteristics shape trust and willingness to follow evidence-based guidance. Understanding these evaluative processes will be critical for ensuring that AI systems support, rather than undermine, evidence-based care and patient-provider relationships.

## Supplementary material

10.2196/97882 Multimedia Appendix 1 Source descriptions and scenario text.

10.2196/97882 Multimedia Appendix 2 Summary of hypotheses and findings.

10.2196/97882 Checklist 1 CHERRIES checklist.

## Abbreviations

AI: artificial intelligence
AI nurse: health care–specialized generative artificial intelligence system framed as an “AI nurse”
CHERRIES: Checklist for Reporting Results of Internet E-Surveys
emmean: estimated marginal mean
M_bene_: mean benevolence score
M_comp_: mean competence score
RQ1: research question 1

## References

[1] Chatterji A, Cunningham T, Deming DJ (2025). How people use ChatGPT. https://www.nber.org/system/files/working_papers/w34255/w34255.pdf.

[2] Mendel T, Singh N, Mann DM, Wiesenfeld B, Nov O (2025). Laypeople’s use of and attitudes toward large language models and search engines for health queries: survey study. J Med Internet Res.

[3] Introducing chatGPT health: a dedicated experience in chatGPT designed for health and wellness. OpenAI.

[4] Rafi MM, Krishnamurthy A, Balu A (2025). Trustworthy LLM-mediated communication: evaluating information fidelity in LLM as a communicator (LAAC) framework in multiple application domains.

[5] Kelly M (2025). Situated epistemic infrastructures: a diagnostic framework for post-coherence knowledge. arXiv.

[6] Nan X, Zhao X, Briones R (2014). Parental cancer beliefs and trust in health information from medical authorities as predictors of HPV vaccine acceptability. J Health Commun.

[7] Sillence E, Briggs P, Harris P, Fishwick L (2006). A framework for understanding trust factors in web-based health advice. Int J Hum Comput Stud.

[8] Zarzeczna N, Hanel PHP, Rutjens BT, Bono SA, Chen YH, Haddock G (2024). Scientists, speak up! Source impacts trust in health advice across five countries. J Exp Psychol Appl.

[9] Hardwig J (1985). Epistemic dependence. J Philos.

[10] Hardwig J (1991). The role of trust in knowledge. J Philos.

[11] Wilholt T (2013). Epistemic trust in science. Br J Philos Sci.

[12] Hendriks F, Kienhues D, Bromme R (2015). Measuring laypeople’s trust in experts in a digital age: the Muenster Epistemic Trustworthiness Inventory (METI). PLoS One.

[13] Landrum AR, Eaves BS, Shafto P (2015). Learning to trust and trusting to learn: a theoretical framework. Trends Cogn Sci.

[14] Pornpitakpan C (2004). The persuasiveness of source credibility: a critical review of five decades’ evidence. J Applied Social Pyschol.

[15] Intemann K (2023). Science communication and public trust in science. Interdiscip Sci Rev.

[16] Rotenberg KJ, Petrocchi S (2018). A longitudinal investigation of trust beliefs in physicians by children with asthma and their mothers: relations with children’s adherence to medical regimes and quality of life. Child Care Health Dev.

[17] Trachtenberg F, Dugan E, Hall MA (2005). How patients’ trust relates to their involvement in medical care. J Fam Pract.

[18] Popowicz DM (2021). Doctor knows best”: on the epistemic authority of the medical practitioner. Philos Med.

[19] Thai CL, Gaysynsky A, Falisi A, Chou WS, Blake K, Hesse BW, Hale TM, Chou W-YS, Cotten SR, Khilnani A (2018). eHealth: Current Evidence, Promises, Perils, and Future Directions.

[20] Many in US consider AI-generated health information useful and reliable. Annenberg Public Policy Center of the University of Pennsylvania.

[21] (2026). 2026 Edelman Trust Barometer: trust amid insularity (global report). https://edl.mn/4stxw32.

[22] Choung H, Seberger JS, David P (2024). When AI is perceived to be fairer than a human: understanding perceptions of algorithmic decisions in a job application vontext. Int J Hum-Comput Interact.

[23] Lu L, Tormala ZL, Duhachek A (2025). How AI sources can increase openness to opposing views. Sci Rep.

[24] Liew TW, Tan SM (2021). Social cues and implications for designing expert and competent artificial agents: a systematic review. Telemat Inform.

[25] Jalbert M, Harris M, Williams L (2025). Who is perceived to be an expert on COVID-19 vaccines on social media?: Biomedical credentials confer expertise, even among vaccine-hesitant and Conservative observers. Inf Commun Soc.

[26] Thon FM, Jucks R (2017). Believing in expertise: how authors’ credentials and language use influence the credibility of online health information. Health Commun.

[27] Alvarado R (2023). What kind of trust does AI deserve, if any?. AI Ethics.

[28] Dlugatch R, Georgieva A, Kerasidou A (2023). Trustworthy artificial intelligence and ethical design: public perceptions of trustworthiness of an AI-based decision-support tool in the context of intrapartum care. BMC Med Ethics.

[29] Guidi S, Reina L, Currò F, Cipriani E, Grassini S, Parlangeli O The influence of framing, domain and task type on trust in AI.

[30] Landrum AR, Lull RB, Akin H, Hasell A, Jamieson KH (2017). Processing the papal encyclical through perceptual filters: Pope Francis, identity-protective cognition, and climate change concern. Cognition.

[31] Kunda Z (1990). The case for motivated reasoning. Psychol Bull.

[32] Petty RE, Cacioppo JT (1986). The elaboration likelihood model of persuasion. Adv Exp Soc Psychol.

[33] Chaiken S (1980). Heuristic versus systematic information processing and the use of source versus message cues in persuasion. J Pers Soc Psychol.

[34] Finucane ML, Alhakami A, Slovic P, Johnson SM (2000). The affect heuristic in judgments of risks and benefits. J Behav Decis Making.

[35] Rimal RN (2001). Perceived risk and self-efficacy as motivators: understanding individuals’ long-term use of health information. J Commun.

[36] Slovic P, Finucane ML, Peters E, MacGregor DG (2004). Risk as analysis and risk as feelings: some thoughts about affect, reason, risk, and rationality. Risk Anal.

[37] Yang JZ (2019). Whose risk? Why did the U.S. public ignore information about the Ebola outbreak?. Risk Anal.

[38] Wilson RS, Arvai JL (2006). When less is more: how affect influences preferences when comparing low and high‐risk options. J Risk Res.

[39] de Hoog N, Stroebe W, de Wit JBF (2008). The processing of fear‐arousing communications: how biased processing leads to persuasion. Soc Influ.

[40] Wedderhoff O, Chasiotis A, Rosman T (2022). When freedom of choice leads to bias: how threat fosters selective exposure to health information. Front Psychol.

[41] Chang DS, Kang OS, Kim HH (2012). Pre-existing beliefs and expectations influence judgments of novel health information. J Health Psychol.

[42] Kahan DM, Braman D, Cohen GL, Gastil J, Slovic P (2010). Who fears the HPV vaccine, who doesn’t, and why? an experimental study of the mechanisms of cultural cognition. Law Hum Behav.

[43] Kahan DM, Jamieson KH, Landrum A, Winneg K (2017). Culturally antagonistic memes and the Zika virus: an experimental test. J Risk Res.

[44] Kim JN, Oh YW, Krishna A (2018). Justificatory information forefending in digital age: self-sealing informational conviction of risky health behavior. Health Commun.

[45] Meppelink CS, Smit EG, Fransen ML, Diviani N (2019). “I was Right about Vaccination”: confirmation bias and health literacy in online health information seeking. J Health Commun.

[46] Claudy MC, Aquino K, Graso M (2022). Artificial intelligence can’t be charmed: the effects of impartiality on laypeople’s algorithmic preferences. Front Psychol.

[47] Jago AS, Laurin K (2022). Assumptions about algorithms’ capacity for discrimination. Pers Soc Psychol Bull.

[48] Fiscella K, Franks P, Clancy CM (1998). Skepticism toward medical care and health care utilization. Med Care.

[49] Levy N (2019). Due deference to denialism: explaining ordinary people’s rejection of established scientific findings. Synthese.

[50] Stavri PZ, Freeman DJ, Burroughs CM (2003). Perception of quality and trustworthiness of Internet resources by personal health information seekers. AMIA Annu Symp Proc.

[51] Reis M, Reis F, Kunde W (2024). Influence of believed AI involvement on the perception of digital medical advice. Nat Med.

[52] Eysenbach G (2004). Improving the quality of Web surveys: the Checklist for Reporting Results of Internet E-Surveys (CHERRIES). J Med Internet Res.

[53] Landrum AR, Verma N, Kehrberg A (2025). Preregistration: asking generative AI health questions. OSF.

[54] Landrum AR, Verma N, Kehrberg A Taking health advice from generative AI. OSF.

[55] McCroskey JC, Teven JJ (1999). Goodwill: a reexamination of the construct and its measurement. Commun Monogr.

[56] Samejima F (2016). Handbook of Item Response Theory.

[57] Greussing E, Guenther L, Baram-Tsabari A (2025). The perception and use of generative AI for science-related information search: insights from a cross-national study. Public Underst Sci.

[58] Shekar S, Pataranutaporn P, Sarabu C, Cecchi GA, Maes P (2025). People overtrust AI-generated medical advice despite low accuracy. NEJM AI.

[59] Qin H, Zhu Y, Jiang Y, Luo S, Huang C (2024). Examining the impact of personalization and carefulness in AI-generated health advice: trust, adoption, and insights in online healthcare consultations experiments. Technol Soc.

[60] Lee ST, Lin J (2016). A self-determination perspective on online health information seeking: the internet vs. face-to-face office visits with physicians. J Health Commun.

[61] Rains SA (2008). Seeking health information in the information age: the role of internet self-efficacy. West J Commun.

[62] Norman CD, Skinner HA (2006). eHealth literacy: essential skills for consumer health in a networked world. J Med Internet Res.

[63] Deci EL, Ryan RM, AW K, ET H (2012). Handbook of Theories of Social Psychology.

