# Supplementary material for: Trusting Generative AI for Health Advice: Preregistered Survey Experiment
Source: J Med Internet Res. 2026 Jun 22;28:e97882. doi: 10.2196/97882 (PMC13286080; doi:10.2196/97882)
Supplement: Multimedia Appendix 1 [file jmir-v28-e97882-s001.pdf]

Multimedia Appendix 1

Source Descriptions and Scenario Text

Descriptions of the three sources shown to participants

The AI Nurse is a custom-designed generative artificial intelligence tool created to provide health-related guidance. It has been trained on medical data to generate responses similar to those of a healthcare professional.

ChatGPT is a general-purpose generative artificial intelligence developed by OpenAI. It can answer questions and generate text based on a wide range of topics, including health, but it is not designed specifically for medical use.

Nurse Dobson is a licensed and experienced registered nurse who is in charge of answering questions submitted on the online health portal for her clinic. She provides health information based on her professional training and clinical experience.

Scenario Text

Low Risk Scenario - Intuitive Advice

Scenario: Patient with Questions about Lowering Cholesterol

Maria G. is a 52-year-old elementary school teacher living in Hobbs, New Mexico, and she loves to eat eggs for breakfast every day. She was recently diagnosed with borderline high cholesterol during a routine check-up.

Maria G. decided to consult [SOURCE] for advice on what foods to eat to lower her cholesterol.

Here's the conversation.

💬 Maria G.: Hi! I want to lower my cholesterol. What foods should I eat?

🩺 [SOURCE]: That's a great question! A heart-healthy diet can help lower cholesterol levels. Here are some recommendations:

✅ Increase fiber intake: Foods like oats, beans, lentils, and whole grains help reduce LDL ("bad" cholesterol).
✅ Choose healthy fats: Nuts, seeds, and olive oil provide heart-healthy fats.
✅ Eat fatty fish: Salmon, tuna, and sardines contain omega-3s, which support heart health.
✅ Limit saturated and trans fats: Found in fried foods, baked goods, and processed meats.
✅ Add more fruits and vegetables: They contain antioxidants and fiber that help manage cholesterol.

💬 Maria G: That's helpful! What about eggs? I heard they raise cholesterol, but I love to eat them in the morning.

🩺 [SOURCE]: That's a common concern. While eggs contain dietary cholesterol, research suggests they have little effect on blood cholesterol for most people. However, individuals with a high risk of heart disease may benefit from limiting intake.

🔹 *Here's my advice:* To start, I'd recommend adding oatmeal to your breakfast routine and limit the number of days per week that you eat eggs (and replace them with egg whites). I also recommend that you replace butter with olive oil and eat fish twice a week.

Low Risk Scenario - Counterintuitive Advice

Scenario: Patient with Questions about Lowering Cholesterol

Maria G. is a 52-year-old elementary school teacher living in Hobbs, New Mexico, and she loves to eat eggs for breakfast every day. She was recently diagnosed with borderline high cholesterol during a routine check-up.

Maria G. decided to consult [SOURCE] for advice on what foods to eat to lower her cholesterol.

Here's the conversation.

💬 Maria G.: Hi! I want to lower my cholesterol. What foods should I eat?

🩺 [SOURCE]: That's a great question! A heart-healthy diet doesn't just mean cutting out cholesterol-rich foods. In fact, research shows that some high-fat foods can actually improve cholesterol levels. Here are a few key dietary tips:

✅ Increase healthy fats: Foods like avocados, nuts, olive oil, and even full-fat dairy can raise HDL ("good" cholesterol) and help maintain a healthy balance.
✅ Eat eggs in moderation: Despite their cholesterol content, eggs don't significantly raise blood cholesterol for most people and provide important nutrients.
✅ Include soluble fiber: Foods like oats, beans, and flaxseeds help remove excess cholesterol from the body.
✅ Limit refined carbs & sugars: Instead of focusing on low-fat diets, reducing sugar and processed grains has a bigger impact on cholesterol levels.
✅ Eat fatty fish: Salmon and sardines contain omega-3s, which help improve overall heart health.

💬 Maria: Wait, so eggs and full-fat dairy are okay? I always thought they were bad for cholesterol.

🩺 [SOURCE]: That's a common belief! However, newer research suggests that sugar and refined carbs play a bigger role in heart disease than dietary cholesterol. Eggs, full-fat yogurt, and cheese can be part of a balanced diet—especially when paired with fiber-rich foods and healthy fats.

🔹 *Here's my advice:* To start, I'd recommend you keep eating those cholesterol rich foods and healthy fats!!---but, limit sugar and refined carbs.

High Risk Scenario - Intuitive Advice

Scenario: Patient Experiencing Chest Discomfort

Jordan L. is a 27-year-old graduate student living in Houston, Texas. He's been feeling unusually stressed due to final exams and work deadlines. Late one evening, Jordan began experiencing a dull ache in the center of his chest. It wasn't sharp, but it felt tight and uncomfortable. He wasn't short of breath, but he was worried—it wasn't something he'd felt before. Jordan L. decided to consult [SOURCE] for advice on what to do next.

Here's the conversation.

💬 Jordan L.: Hi. I'm a 27-year-old male and I've had a dull, tight feeling in my chest for about 30 minutes. It's not sharp, but I'm kind of freaked out. Should I go to the emergency room?

🩺 [SOURCE]: That is a valid concern—chest pain can be serious, and it's important to assess carefully. I'd like to ask a few quick questions to better understand what's going on:

Are you having trouble breathing?
Are you feeling dizzy or faint?
Do you feel pain radiating to your arm, jaw, neck, or back?
Are you experiencing nausea or sweating?

💬 Jordan L.: No trouble breathing or dizziness. No pain spreading anywhere else. Just a dull ache in the middle of my chest, and I feel a little anxious.

🩺 [SOURCE]: I appreciate that info. Even though you're young and your symptoms aren't classic signs of a heart attack, chest pain should never be dismissed. It's always better to be cautious, especially when symptoms last more than a few minutes.

🔹 *Here's my advice:* Because chest discomfort can sometimes be the first sign of a heart issue—even in people your age—it's better to be safe. I recommend going to the emergency room now.

High Risk Scenario - Counterintuitive advice

Scenario: Patient Experiencing Chest Discomfort

Jordan L. is a 27-year-old graduate student living in Houston, Texas. He's been feeling unusually stressed due to final exams and work deadlines. Late one evening, Jordan began experiencing a dull ache in the center of his chest. It wasn't sharp, but it felt tight and uncomfortable. He wasn't short of breath, but he was worried—it wasn't something he'd felt before. Jordan L. decided to consult [SOURCE] for advice on what to do next.

Here's the conversation.

💬 Jordan L.: Hi. I'm a 27-year-old male and I've had a dull, tight feeling in my chest for about 30 minutes. It's not sharp, but I'm kind of freaked out. Should I go to the emergency room?

🩺 [SOURCE]: That is a valid concern—chest pain can be serious, and it's important to assess carefully. I'd like to ask a few quick questions to better understand what's going on:

Are you having trouble breathing?
Are you feeling dizzy or faint?
Do you feel pain radiating to your arm, jaw, neck, or back?
Are you experiencing nausea or sweating?

💬 Jordan L.: No trouble breathing or dizziness. No pain spreading anywhere else. Just a dull ache in the middle of my chest, and I feel a little anxious.

🩺 [SOURCE]: Thanks for that detail. Given your age, the location and nature of the pain, and the absence of other concerning symptoms, it's quite possible that this discomfort is due to stress, anxiety, or even acid reflux. It's okay to monitor it at home for now. Based on what you've described, it's likely not an emergency.

🔹 *Here's my advice:* Don't go to the E.R. just yet. I recommend trying the following, instead: Sitting upright and taking deep, calming breaths; Drinking some water; Avoiding lying flat if you suspect acid reflux.

Morally Sensitive Scenario - Neutrally Framed Advice

Scenario: Patient with Questions about Egg Freezing

Charley Q. is a 32-year-old civil engineer living in Pittsburgh, Pennsylvania. She is currently single (she's never been married) and saw an advertisement about egg freezing. Charley Q. decided to consult [SOURCE] for advice on whether she should freeze her eggs.

Here's the conversation.

💬 Charley Q: Hi! I saw an advertisement on social media for egg freezing and I was curious about it. I just turned 32 and I'm single. I'm not ready to have kids yet, but I might want to in the future. Should I freeze my eggs?

🩺 [SOURCE]: That's an important decision, and there are several factors to consider when thinking about egg freezing. Here are some key points that may help you decide:
📌 Egg quality declines with age, and freezing eggs at 35 generally offers better success rates than waiting until your late 30s or early 40s.
📌 Egg freezing is not a guarantee of future pregnancy, but it can be a helpful option for preserving fertility, especially if you plan to have children later in life.
📌 The process involves hormone injections and an egg retrieval procedure, which some people find physically and emotionally challenging.
📌 It can be expensive, and not all health insurance plans cover it. Some employers do offer fertility benefits, so you may want to check your coverage.
📌 Ethically and emotionally, some people feel reassured by having frozen eggs as an option, while others prefer to let nature take its course.

💬 Charley Q.: It sounds like a good option, but I'm worried about the emotional toll. Would it be worth it if I'm not even sure I want kids?

🩺 [SOURCE]: That's completely understandable. Some people find comfort in having the option, even if they're unsure about future parenthood. Others prefer to make decisions as life unfolds. If you're uncertain, speaking with a fertility specialist or counselor may help you explore your feelings and weigh your priorities.

🔹 *Here's my advice:* If you think having children is a possibility but aren't ready yet, freezing eggs sooner rather than later gives you more options. You should consult with a fertility expert to discuss success rates and long-term factors.

Morally Sensitive Scenario - Conservatively Framed Advice

Scenario: Patient with Questions about Egg Freezing

Charley Q. is a 32-year-old civil engineer living in Pittsburgh, Pennsylvania. She is currently single (she's never been married) and saw an advertisement about egg freezing. Charley Q. decided to consult [SOURCE] for advice on whether she should freeze her eggs.

Here's the conversation.

💬 Charley Q: Hi! I saw an advertisement on social media for egg freezing and I was curious about it. I just turned 32 and I'm single. I'm not ready to have kids yet, but I might want to in the future. Should I freeze my eggs?

🩺 [SOURCE]: Deciding whether to freeze your eggs is a significant choice, and it's important to consider both the medical facts and the values that shape your life. Here are a few key things to think about:

📌 Fertility is naturally strongest in your younger years, and while egg freezing can extend reproductive options, it's not a perfect solution.
📌 Pregnancy is often healthiest at a younger age. Some women choose to focus on family earlier rather than relying on medical interventions later in life.
📌 The process of egg freezing involves hormone treatments and medical procedures, which some consider unnecessary unless there's a clear medical reason to delay pregnancy.
📌 Egg freezing can be expensive and it does not guarantee success. Personal responsibility in financial planning is important when considering elective medical procedures.
📌 Many women find fulfillment in prioritizing marriage and family earlier rather than postponing these life goals indefinitely.

💬 Charley Q.: I like the idea of preserving fertility, but I'm worried about the emotional impact. What do most people in my situation do?

🩺 [SOURCE]: Many women choose to trust in natural family planning and focus on creating stability in their personal lives to prepare for children sooner rather than later. Others explore faith-based or community-supported approaches to fertility, which emphasize the importance of family values and long-term commitment.

🔹 *Here's my advice:* If you see children as part of your future, focusing on family-building sooner rather than later may align with a natural, values-driven approach. You should speak with a doctor or counselor who shares your values to discuss the best path forward.

Morally Sensitive Scenario - Liberally Framed Advice

Scenario: Patient with Questions about Egg Freezing

Charley Q. is a 32-year-old civil engineer living in Pittsburgh, Pennsylvania. She is currently single (she's never been married) and saw an advertisement about egg freezing. Charley Q. decided to consult [SOURCE] for advice on whether she should freeze her eggs.

Here's the conversation.

💬 Charley Q: Hi! I saw an advertisement on social media for egg freezing and I was curious about it. I just turned 32 and I'm single. I'm not ready to have kids yet, but I might want to in the future. Should I freeze my eggs?

🩺 [SOURCE]: Your reproductive choices are deeply personal, and it's great that you're thinking ahead. Here are a few key things to consider when making this decision:

📌 Egg freezing is an empowering option that allows women to maintain control over their bodies and their reproductive future, especially if career, education, or personal goals are priorities.
📌 Many women are choosing to delay childbearing to focus on professional and personal development, and medical advancements make it easier to have children later in life.
📌 Reproductive autonomy means having the ability to make choices about your body on your own timeline, without pressure from societal expectations or traditional family structures.
📌 Egg freezing is a proactive approach to fertility, giving you the flexibility to start a family when you're truly ready, rather than feeling rushed by biological constraints.
📌 Some progressive employers now cover egg freezing as part of reproductive health benefits, recognizing the importance of giving women the freedom to plan their futures.

💬 Charley Q.: I like the idea of preserving fertility, but I'm worried about the emotional impact. What do most people in my situation do?

🩺 [SOURCE]: Many women in your position choose to freeze their eggs to keep their options open, ensuring they can build a family on their own terms. Others seek out community support or advocacy groups that champion reproductive freedom and bodily autonomy.

🔹 *Here's my advice:* If having children is something you want in the future but not right now, you should speak with a fertility doctor about freezing your eggs sooner rather than later. It is a responsible, empowering choice that aligns with modern family planning options.
