# Supplementary material for: Trusting Generative AI for Health Advice: Preregistered Survey Experiment
Source: J Med Internet Res. 2026 Jun 22;28:e97882. doi: 10.2196/97882 (PMC13286080; doi:10.2196/97882)
Supplement: Multimedia Appendix 2 [file jmir-v28-e97882-s002.pdf]

Multimedia Appendix 2

Summary of hypotheses and findings.

Hypothesis	Prediction	Outcome	Supported?
H1a	Human sources would be perceived as more competent than AI sources.	Human nurse was rated significantly more competent than both AI Nurse and ChatGPT. No difference between the two AI sources.	Supported
H1b	Human sources would be perceived as more benevolent/trustworthy than AI sources.	Human nurse was rated significantly more benevolent than both AI sources. No difference between the two AI sources.	Supported
H1c	Advice from a human nurse would be perceived as more credible than advice from AI sources.	Advice from the human nurse was rated as more credible than advice from both AI Nurse and ChatGPT.	Supported
H1d	Advice from a human nurse would be seen as more worthy of being followed ("Take Advice") than advice from AI sources.	Participants agreed more strongly that the character should follow the human nurse's advice than advice from either AI source.	Supported
H2a	Intuitive advice would be judged as more credible than counterintuitive advice.	Intuitive advice was rated as more credible and more worthy of following than counterintuitive advice.	Supported
H2b	The difference between intuitive and counterintuitive advice would be amplified in high-risk contexts.	Significant interaction between advice intuitiveness and risk level: counterintuitive advice suffered a larger credibility penalty in the high-risk scenario.	Supported
H3	Ideological framing would shape perceptions of advice bias and credibility in the morally sensitive scenario.	Advice framing significantly influenced perceived bias, credibility, and willingness to follow advice. Conservative framing was viewed less favorably than neutral or liberal framing.	Supported
H4	AI sources would be perceived as less biased than the human nurse.	No significant effect of source on perceived bias and no source interactions.	Not Supported
RQ1a	Medical skepticism would moderate credibility evaluations of human and AI sources.	Medical skepticism interacted with source evaluations: higher skepticism increased perceived competence of the AI Nurse and decreased evaluations of the human nurse.	Partial support - Some moderation effects observed
RQ1b	Prior experience with AI tools would moderate credibility evaluations of human and AI sources.	Greater AI experience predicted higher competence and benevolence ratings overall, but interactions with source were not significant.	Moderation not supported
