# Supplementary material for: Trusting Generative AI for Health Advice: Preregistered Survey Experiment
Source: J Med Internet Res. 2026 Jun 22;28:e97882. doi: 10.2196/97882 (PMC13286080; doi:10.2196/97882)
Supplement: Checklist 1 [file jmir-v28-e97882-s003.pdf]

Multimedia Appendix 3

Checklist for Reporting Results of Internet E-Surveys (CHERRIES)

Checklist Item	***Explanation***	***Our Study***	***Page Number***
Describe survey design	Describe target population, sample frame. Is the sample a convenience sample? (In "open" surveys this is most likely.)	U.S. National Adult Sample (18 and older) fielded by CINT Theorem.	7—9
IRB approval	Mention whether the study has been approved by an IRB.	The study was approved and categorized as exempt by the Arizona State University Internal Review Board on October 3, 2024 (STUDY00020970).	7
Informed consent	Describe the informed consent process. Where were the participants told the length of time of the survey, which data were stored and where and for how long, who the investigator was, and the purpose of the study?	The first page of the online survey provided information about the study including that participation is voluntary, they have to pass the attention check questions in order to progress to the end of the survey, and no personal information would be collected. Surveys on CINT Theorem must be 15 minutes or less (median). Median time for completion for our survey was 11.97 minutes.	8
Data protection	If any personal information was collected or stored, describe what mechanisms were used to protect unauthorized access.	No personal or identifying information was collected or stored.	7
Development and testing	State how the survey was developed, including whether the usability and technical functionality of the electronic questionnaire had been tested before fielding the questionnaire.	Usability and technical functionality of the survey was not tested beyond the metrics provided by Qualtrics during survey creation.	8
Open survey versus closed survey	An "open survey" is a survey open for each visitor of a site, while a closed survey is only open to a sample which the investigator knows (password-protected survey).	It was a closed survey, by invitation only, that was fielded by CINT Theorem.	7—8
Contact mode	Indicate whether or not the initial contact with the potential participants was made on the Internet. (Investigators may also send out questionnaires by mail and allow for Web-based data entry.)	CINT Theorem handled recruitment via their relationship with additional internet panels.	8
Advertising the survey	How/where was the survey announced or advertised? Some examples are offline media (newspapers), or online (mailing lists – If yes, which ones?) or banner ads (Where were these banner ads posted and what did they look like?). It is important to know the wording of the announcement as it	CINT Theorem handled recruitment via their relationship with additional internet panels. We did not recruit participants.	8

Multimedia Appendix 3

	will heavily influence who chooses to participate. Ideally the survey announcement should be published as an appendix.		
Web/E-mail	State the type of e-survey (eg, one posted on a Web site, or one sent out through e-mail). If it is an e-mail survey, were the responses entered manually into a database, or was there an automatic method for capturing responses?	CINT Theorem handled recruitment. Our survey was administered via Qualtrics Survey Software. Responses were captured by Qualtrics and stored by Qualtrics.	8
Context	Describe the Web site (for mailing list/newsgroup) in which the survey was posted. What is the Web site about, who is visiting it, what are visitors normally looking for? Discuss to what degree the content of the Web site could pre-select the sample or influence the results. For example, a survey about vaccination on a anti-immunization Web site will have different results from a Web survey conducted on a government Web site	CINT Theorem handled recruitment via their relationship with additional internet panels. We did not recruit participants.	8
Mandatory/voluntary	Was it a mandatory survey to be filled in by every visitor who wanted to enter the Web site, or was it a voluntary survey?	The survey was voluntary. This was specified on the Consent page.	8
Incentives	Were any incentives offered (eg, monetary, prizes, or non-monetary incentives such as an offer to provide the survey results)?	Participants were compensated in line with their agreements with their survey panel providers.	8
Time/Date	In what timeframe were the data collected?	July 14 to 15, 2025	8
Randomization of items or questionnaires	To prevent biases items can be randomized or alternated.	We used the randomizing function in Qualtrics to randomize condition assignment, scenario order, and item order.	8—10
Adaptive questioning	Use adaptive questioning (certain items, or only conditionally displayed based on responses to other items) to reduce number and complexity of the questions.	We did not use adaptive questioning.	N/A
Number of Items	What was the number of questionnaire items per page? The number of items is an important factor for the completion rate.	This depended on the page and the question types. Matrix-style questions had more items per page than standard multiple choice items. Five pages of the survey had only one question.	Osf.io site
Number of screens (pages)	Over how many pages was the questionnaire distributed? The number of items is an important factor for the completion rate.	16 pages	Osf.io site

Multimedia Appendix 3

Completeness check	It is technically possible to do consistency or completeness checks before the questionnaire is submitted. Was this done, and if "yes", how (usually JAVAScript)? An alternative is to check for completeness after the questionnaire has been submitted (and highlight mandatory items). If this has been done, it should be reported. All items should provide a non-response option such as "not applicable" or "rather not say", and selection of one response option should be enforced.	We included "request response" validation on each page so that if a question was not completed, participants were told that there were unanswered questions and asked to respond. They could, however, choose to submit without answering the questions.	
Review step	State whether respondents were able to review and change their answers (eg, through a Back button or a Review step which displays a summary of the responses and asks the respondents if they are correct).	No, participants were not given the opportunity to change their answers.	
Unique site visitor	If you provide view rates or participation rates, you need to define how you determined a unique visitor. There are different techniques available, based on IP addresses or cookies or both.	We selected a security option in Qualtrics survey software that is supposed to prevent multiple submissions with duplicate detection as well as scan for bots. However, since we went through CINT Theorem, participants were signed in to complete the survey through their unique response id numbers that also allowed the survey panels to know which participants completed the survey and provide them with the agreed upon incentive.	
View rate (Ratio of unique survey visitors/unique site visitors)	Requires counting unique visitors to the first page of the survey, divided by the number of unique site visitors (not page views!). It is not unusual to have view rates of less than 0.1 % if the survey is voluntary.	We do not have this data.	
Participation rate (Ratio of unique visitors who agreed to participate/unique first survey page visitors)	Count the unique number of people who filled in the first survey page (or agreed to participate, for example by checking a checkbox), divided by visitors who visit the first page of the survey (or the informed consents page, if present). This can also be called "recruitment" rate.	We do not have this data.	
Completion rate (Ratio of users who finished the	The number of people submitting the last questionnaire page, divided by the number of people who agreed to participate (or submitted the first survey page). This is only relevant if there is a separate "informed consent" page or if	97% (but this does not include participants who were booted out of the survey due to inaccurately answering the attention check question)	

survey/users who agreed to participate)	the survey goes over several pages. This is a measure for attrition. Note that "completion" can involve leaving questionnaire items blank. This is not a measure for how completely questionnaires were filled in. (If you need a measure for this, use the word "completeness rate".)		
Cookies used	Indicate whether cookies were used to assign a unique user identifier to each client computer. If so, mention the page on which the cookie was set and read, and how long the cookie was valid. Were duplicate entries avoided by preventing users access to the survey twice; or were duplicate database entries having the same user ID eliminated before analysis? In the latter case, which entries were kept for analysis (eg, the first entry or the most recent)?	NA	
IP check	Indicate whether the IP address of the client computer was used to identify potential duplicate entries from the same user. If so, mention the period of time for which no two entries from the same IP address were allowed (eg, 24 hours). Were duplicate entries avoided by preventing users with the same IP address access to the survey twice; or were duplicate database entries having the same IP address within a given period of time eliminated before analysis? If the latter, which entries were kept for analysis (eg, the first entry or the most recent)?	We relied on Qualtrics' duplicate detection function.	
Log file analysis	Indicate whether other techniques to analyze the log file for identification of multiple entries were used. If so, please describe.	N/A	
Registration	In "closed" (non-open) surveys, users need to login first and it is easier to prevent duplicate entries from the same user. Describe how this was done. For example, was the survey never displayed a second time once the user had filled it in, or was the username stored together with the survey results and later eliminated? If the latter, which entries were kept for analysis (eg, the first entry or the most recent)?	This was handled by CINT Theorem.	

Multimedia Appendix 3

Handling of incomplete questionnaires	Were only completed questionnaires analyzed? Were questionnaires which terminated early (where, for example, users did not go through all questionnaire pages) also analyzed?	Participants were listwise deleted from analyses that used items/questions for which they did not have responses.	
Questionnaires submitted with an atypical timestamp	Some investigators may measure the time people needed to fill in a questionnaire and exclude questionnaires that were submitted too soon. Specify the timeframe that was used as a cut-off point, and describe how this point was determined.	N/A	
Statistical correction	Indicate whether any methods such as weighting of items or propensity scores have been used to adjust for the non-representative sample; if so, please describe the methods.	N/A	

This checklist has been modified from Eysenbach G. Improving the quality of Web surveys: the Checklist for Reporting Results of Internet E-Surveys (CHERRIES). J Med Internet Res. 2004 Sep 29;6(3):e34 [erratum in J Med Internet Res. 2012; 14(1): e8.]. Article available at https://www.jmir.org/2004/3/e34/; erratum available https://www.jmir.org/2012/1/e8/. Copyright ©Gunther Eysenbach. Originally published in the Journal of Medical Internet Research, 29.9.2004 and 04.01.2012.
